# Physical Literacy and College Adjustment Among Chinese International Students Who Regularly Participate in Basketball in South Korea: The Mediating Role of Well-Being

**DOI:** 10.3390/bs16071241

**Published:** 2026-07-21

**Authors:** Shuangrui Liu, Seungwoo Choi, Ansu Lee

**Affiliations:** Department of Physical Education, Kyungpook National University, Daegu 41566, Republic of Korea; lsr980324@knu.ac.kr

**Keywords:** physical literacy, well-being, college adjustment, Chinese international students, psychosocial adaptation

## Abstract

Chinese international students studying in South Korea may experience academic, sociocultural, and psychological challenges that may undermine well-being and successful adaptation to university life. Physical literacy, which encompasses motivation, confidence, physical competence, knowledge, and understanding related to physical activity, may represent a behavioral and psychosocial resource that supports adaptive functioning. This cross-sectional study examined the relationships among perceived physical literacy, well-being, and college adjustment among 288 Chinese international students in South Korea who participated in basketball at least once per week. Physical literacy was assessed using the Perceived Physical Literacy Instrument, well-being using the Mental Health Continuum–Short Form, and college adjustment using the Student Adaptation to College Questionnaire-25. Confirmatory factor analysis and structural equation modeling were conducted, and the mediating effect of well-being was tested using bootstrapping with 2000 resamples. Physical literacy was positively associated with well-being (β = 0.305, *p* < .001), well-being was positively associated with college adjustment (β = 0.358, *p* < .001), and physical literacy was positively associated with college adjustment (β = 0.180, *p* = .002). The indirect effect of physical literacy on college adjustment through well-being was significant (β = 0.109, *p* = .001), indicating partial mediation. These findings suggest that, within this sample of Chinese international students who participated in basketball at least once per week, physical literacy may be relevant to college adjustment as a behavioral and psychosocial resource, partly through well-being.

## 1. Introduction

International students represent a rapidly growing population in higher education and frequently encounter academic, social, linguistic, and cultural challenges during their transition to university life in a host country. These challenges may negatively affect psychological well-being (WB), social integration, academic engagement, and overall adaptation to the university environment (Glass et al., 2014; Sawir et al., 2008; Smith & Khawaja, 2011; Zhang & Goodson, 2011). Recent research has further suggested that international student adjustment is shaped not only by individual coping resources but also by campus-level factors, including social support, campus resources, inclusive environments, cultural diversity, and opportunities for social engagement (Brunsting et al., 2018; Huang et al., 2024). Although international student adjustment has often been examined from educational and institutional perspectives, it can also be understood as a psychosocial adaptation process that involves managing unfamiliar cultural environments, developing new social relationships, and coping with multiple sources of stress (Brunsting et al., 2018; Smith & Khawaja, 2011; Zhang & Goodson, 2011). Consequently, identifying behavioral and psychosocial resources that facilitate successful adaptation has become increasingly important in behavioral science research (Brunsting et al., 2018; Huang et al., 2024).

College adjustment (CA) refers to the extent to which students successfully adapt to the academic, social, emotional, and institutional demands of university life (Baker & Siryk, 1984). Successful adjustment has been associated with academic achievement, persistence, retention, life satisfaction, and psychological functioning (Pritchard & Wilson, 2003). For international students, adjustment extends beyond academic adaptation and includes sociocultural integration, emotional coping, interpersonal relationship development, and attachment to the host institution (Brunsting et al., 2018; Smith & Khawaja, 2011; Zhang & Goodson, 2011). Conversely, difficulties in adjustment may contribute to loneliness, psychological distress, and reduced engagement with university life (Glass et al., 2014; Sawir et al., 2008; Smith & Khawaja, 2011; Zhang & Goodson, 2011). Given the complexity of this adaptation process, it is important to identify individual resources that may promote positive functioning during cultural transition (Brunsting et al., 2018; Credé & Niehorster, 2012).

From a behavioral science perspective, physical activity (PA) and sport participation represent more than health-related behaviors. Participation in organized PA provides opportunities for competence development, social interaction, emotional regulation, identity formation, and meaningful engagement with others (Bailey, 2006; Biddle & Asare, 2011; Eime et al., 2013; Lubans et al., 2016). These experiences may contribute to psychological and social functioning that extends beyond physical health outcomes (Eime et al., 2013; Lubans et al., 2016; Opstoel et al., 2020). For international students in particular, sport and PA settings may serve as important contexts for social integration, intercultural interaction, and the development of resources that support successful adaptation to university life (Glass et al., 2014; Huang et al., 2024).

The potential contribution of PA and sport participation to student adjustment may be understood through a biopsychosocial perspective. At the biological level, regular PA may support physical vitality, stress regulation, and cognitive functioning, which may help students manage the demands of university life (Biddle & Asare, 2011; Lubans et al., 2016). At the psychological level, participation may strengthen confidence, perceived competence, emotional regulation, resilience, and self-efficacy (Lubans et al., 2016; Opstoel et al., 2020). At the social level, organized sport may provide opportunities for peer interaction, social support, belonging, and intercultural engagement (Eime et al., 2013; Glass et al., 2014; Opstoel et al., 2020). These interconnected biological, psychological, and social processes may help explain why movement-related resources are associated with both WB and adaptation.

Within this biopsychosocial context, physical literacy (PL) may represent an important individual resource that shapes how people engage with and benefit from PA and sport. PL has emerged as a holistic construct encompassing motivation, confidence, physical competence, knowledge, and understanding that support lifelong engagement in PA (Whitehead, 2019; Whitehead et al., 2018). Contemporary perspectives conceptualize PL as an integrated construct involving physical, cognitive, affective, and behavioral dimensions of human functioning (Carl et al., 2022; Dudley, 2015; Edwards et al., 2017). Increasing evidence suggests that PL is associated not only with PA participation but also with mental health, resilience, flourishing, and broader indicators of WB (Cairney et al., 2019; Caldwell et al., 2020; Chen, 2015; Ma et al., 2021). Rather than representing physical competence alone, PL may be conceptualized as a multidimensional behavioral and psychosocial resource that supports meaningful participation and engagement across diverse movement contexts (Cairney et al., 2019; Edwards et al., 2017; Whitehead et al., 2018). Individuals with higher levels of PL may be better equipped to participate confidently in movement-related experiences, establish social connections, and derive psychological benefits from PA contexts (Cairney et al., 2019; Ma et al., 2021; Whitehead et al., 2018). These characteristics suggest that PL may contribute to psychosocial adaptation, although this possibility has received limited empirical attention among international students.

WB is a multidimensional construct reflecting positive functioning across emotional, psychological, and social domains (Keyes, 2002). Emotional WB encompasses positive affect and satisfaction with life, whereas psychological WB reflects positive individual functioning, including autonomy, purpose in life, personal growth, and effective management of one’s environment. Social WB concerns individuals’ perceived integration, contribution, acceptance, and functioning within society (Keyes, 2002; Ryff, 2014). These dimensions are particularly relevant to international students, who must cope with academic and cultural stressors while developing new interpersonal relationships and learning to function within an unfamiliar social and institutional environment. Higher levels of WB may support effective coping, resilience, interpersonal functioning, and engagement with university life, whereas lower levels of WB may increase vulnerability to social isolation, psychological distress, and adjustment difficulties (Brunsting et al., 2018; Credé & Niehorster, 2012; Diener et al., 2018; Keyes, 2002).

The potential relationships among PL, WB, and CA may be understood through Self-Determination Theory (SDT), which proposes that psychological functioning and WB are supported when the basic psychological needs for competence, autonomy, and relatedness are satisfied (Deci & Ryan, 2000; Ntoumanis & Moller, 2025). PL may facilitate participation experiences that satisfy these psychological needs. Individuals with higher levels of PL may feel more competent in PA settings, more confident and self-directed in initiating and maintaining participation, and better able to engage in meaningful social interactions (Cairney et al., 2019; Whitehead et al., 2018). Such experiences may support emotional, psychological, and social WB by strengthening competence, autonomy, and relatedness (Deci & Ryan, 2000; Ntoumanis & Moller, 2025). In turn, higher levels of WB may facilitate successful adjustment by supporting emotional regulation, interpersonal functioning, resilience, and effective coping with academic and sociocultural challenges (Credé & Niehorster, 2012; Huang et al., 2024; Keyes, 2002). Consequently, WB may represent an important psychological mechanism underlying the association between PL and successful adaptation among international students (Diener et al., 2018; Keyes, 2002; Ma et al., 2021).

Despite growing interest in PL, WB, and CA, limited research has examined these constructs within a single explanatory framework among international students. Previous studies have reported positive associations between PL and WB and between WB and adaptation-related outcomes (Credé & Niehorster, 2012; Ma et al., 2021). However, the relationship between PL and CA remains underexplored, and little is known about whether WB serves as a psychological mechanism linking the two constructs. Although recent studies have highlighted the importance of supportive campus environments for international student adjustment (Brunsting et al., 2018; Huang et al., 2024), relatively little attention has been paid to individual movement-related resources that may promote psychological functioning and adaptation. Examining these relationships may provide a more comprehensive understanding of psychosocial adaptation among international students, particularly those who regularly participate in organized PA or sport.

Therefore, this study examined the relationships among perceived PL, WB, and CA among Chinese international students in South Korea who participated in basketball at least once per week. Specifically, this study investigated whether WB mediates the relationship between PL and CA. Based on previous literature and SDT, we hypothesized that PL would be positively associated with WB (H1), WB would be positively associated with CA (H2), PL would be positively associated with CA (H3), and WB would mediate the relationship between PL and CA (H4).

## 2. Materials and Methods

### 2.1. Study Design

**Figure 1:**
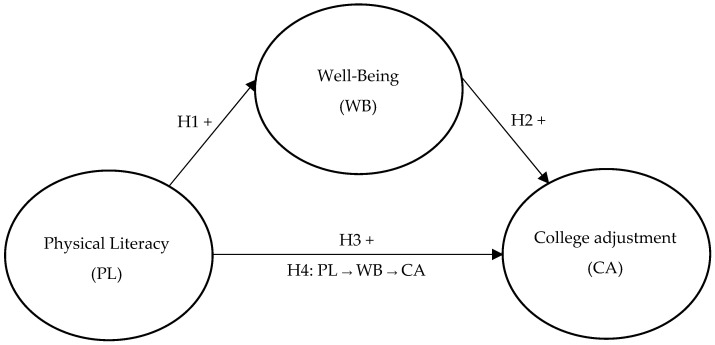
Hypothesized mediation model linking physical literacy, well-being, and college adjustment.

This study employed a cross-sectional quantitative design to examine the relationships among perceived PL, WB, and CA among Chinese international students in South Korea who participated in basketball at least once per week. A cross-sectional design was considered appropriate because the study aimed to examine associations among the study variables at a single point in time rather than changes over time (X. Wang & Cheng, 2020). Structural equation modeling (SEM) was used to test the hypothesized direct relationships among PL, WB, and CA (H1–H3) and the indirect effect of PL on CA through WB (H4). The hypothesized model is presented in Figure 1.

### 2.2. Participants and Procedure

Participants were Chinese international students aged 18 years or older who were enrolled at South Korean universities and participated in basketball at least once per week. Chinese international students were selected because they constitute a major international student group in South Korea and often experience academic, linguistic, sociocultural, and psychosocial challenges during adaptation to university life in a host country (Andrade, 2006; Smith & Khawaja, 2011; Zhang & Goodson, 2011). Focusing on this group allowed the study to examine the relationships among PL, WB, and CA within a relatively specific international student context.

Basketball participants were recruited because university basketball activities represent a regular, group-based PA context that involves physical engagement, teamwork, communication, and peer interaction. Previous studies have suggested that participation in sport and recreational activities can facilitate social interaction, interpersonal development, and psychosocial adjustment (Eime et al., 2013; Glass et al., 2014; Opstoel et al., 2020). This sampling criterion was therefore consistent with the purpose of the study, which was to examine PL as a behavioral and psychosocial resource within a socially embedded movement context. Participation in basketball at least once per week was used as an inclusion criterion to ensure that respondents had ongoing exposure to a shared PA setting rather than only occasional or incidental sport participation.

Data were collected in November 2025 using an online questionnaire administered through Wenjuanxing. Recruitment announcements were distributed via WeChat and KakaoTalk, which are widely used among Chinese international students in South Korea. Prior to participation, respondents received an online information sheet describing the study purpose, eligibility criteria, confidentiality procedures, voluntary participation, and the right to withdraw at any time. Electronic informed consent was obtained before survey completion. No incentives were provided.

A total of 294 students completed the survey. After excluding six responses due to incomplete data or inattentive responding, the final sample consisted of 288 participants (155 males, 53.8%; 133 females, 46.2%). Regarding academic year, 21.2% were first-year undergraduate students, 20.8% were second-year undergraduate students, 21.2% were third-year undergraduate students, 19.4% were fourth-year undergraduate students, 11.1% were master’s students, and 6.2% were doctoral students. The largest proportion had studied in South Korea for 6 months to less than 1 year (34.4%), and most participated in basketball two or three times per week (57.3%).

### 2.3. Measures

Because the participants were Chinese international students, all instruments were administered in Chinese. The measurement instruments were translated using a forward–backward translation procedure. First, the original items were translated into Chinese by bilingual researchers familiar with physical education, physical literacy, and student adjustment research. Second, the translated items were independently back-translated into English. Third, discrepancies between the original and back-translated versions were reviewed by the research team, with particular attention to conceptual equivalence, clarity, and cultural appropriateness. Minor wording adjustments were made to ensure that the items were understandable for Chinese international students while preserving the conceptual meaning of the original instruments.

#### 2.3.1. Measurement of Physical Literacy

Perceived PL was assessed using the nine-item Perceived Physical Literacy Instrument (PPLI) developed by Sum et al. (2016). The instrument consists of three dimensions: sense of self and self-confidence, self-expression and communication with others, and knowledge and understanding. Responses were recorded on a five-point Likert scale ranging from 1 (strongly disagree) to 5 (strongly agree), with higher scores indicating higher levels of perceived PL. Cronbach’s α coefficients were 0.844 for sense of self and self-confidence, 0.767 for self-expression and communication with others, and 0.826 for knowledge and understanding. The overall scale demonstrated excellent internal consistency (α = 0.930).

#### 2.3.2. Measurement of College Adjustment

CA was measured using the 25-item Student Adaptation to College Questionnaire (SACQ-25) developed by Baker and Siryk (1984) and adapted for the Korean context by Lee (2000). The instrument measures five dimensions: academic adjustment, social adjustment, emotional adjustment, physical adjustment, and attachment to the university. Responses were rated on a five-point Likert scale, with higher scores indicating better adjustment to university life. Cronbach’s α coefficients ranged from 0.862 to 0.883 across the five subscales, and the overall reliability coefficient was 0.973.

#### 2.3.3. Measurement of Well-Being

WB was assessed using the Mental Health Continuum–Short Form (MHC-SF) developed by Keyes (2002). The instrument consists of 14 items measuring emotional, social, and psychological WB. Responses were recorded on a six-point frequency scale ranging from 0 (never) to 5 (every day), with higher scores indicating higher levels of WB. Cronbach’s α coefficients were 0.877 for emotional WB, 0.927 for social WB, and 0.940 for psychological WB. The overall scale demonstrated excellent internal consistency (α = 0.973).

### 2.4. Data Analysis

Data were analyzed using SPSS version 30.0 (IBM Corp., Armonk, NY, USA) and AMOS version 27.0 (IBM Corp., Armonk, NY, USA). Descriptive statistics, including means, standard deviations, skewness, and kurtosis, were calculated to examine the distributional characteristics of the study variables. Internal consistency reliability was evaluated using Cronbach’s α coefficients, and Pearson correlation analyses were conducted to examine bivariate associations among PL, WB, and CA.

Confirmatory factor analysis (CFA) was conducted to evaluate the measurement models before the structural model was tested, consistent with contemporary structural equation modeling practice (Kline, 2023). Model fit was assessed using the chi-square statistic relative to degrees of freedom (χ^2^/df), comparative fit index (CFI), Tucker–Lewis index (TLI), root mean square error of approximation (RMSEA), and standardized root mean square residual (SRMR). Convergent validity was evaluated using composite reliability (CR) and average variance extracted (AVE), and discriminant validity was assessed using the Fornell–Larcker criterion.

SEM was then performed to test the hypothesized relationships among PL, WB, and CA. The mediating effect of WB was examined using bootstrapping with 2000 resamples. Statistical significance was set at *p* < .05.

## 3. Results

### 3.1. Descriptive Statistics and Correlations

Descriptive statistics and correlations among the main study variables are presented in Table 1. The mean scores were 3.70 (SD = 0.95) for PL, 4.69 (SD = 1.18) for WB, and 3.71 (SD = 0.90) for CA. Skewness and kurtosis values were within acceptable ranges, indicating no substantial deviation from normality.

Pearson correlation analysis showed that PL was positively correlated with WB (r = 0.298, *p* < .001) and CA (r = 0.275, *p* < .001). WB was also positively correlated with CA (r = 0.400, *p* < .001). These results provided preliminary support for the hypothesized positive associations among the study variables.

### 3.2. Measurement Models

CFA was conducted to evaluate the measurement models before testing the structural model. The three-factor PL model showed good fit, χ^2^(24) = 43.459, χ^2^/df = 1.811, CFI = 0.988, TLI = 0.982, RMSEA = 0.053, and SRMR = 0.023. The three-factor WB model also showed good fit, χ^2^(74) = 133.113, χ^2^/df = 1.799, CFI = 0.986, TLI = 0.982, RMSEA = 0.053, and SRMR = 0.019. The five-factor CA model demonstrated acceptable fit, χ^2^(265) = 504.824, χ^2^/df = 1.905, CFI = 0.957, TLI = 0.951, RMSEA = 0.056, and SRMR = 0.030.

Convergent validity was supported, with CR values ranging from 0.769 to 0.940 and AVE values ranging from 0.526 to 0.722. Discriminant validity was evaluated using the Fornell–Larcker criterion. As shown in Table 2, the square root of the AVE for each construct exceeded its correlations with other constructs, supporting discriminant validity.

### 3.3. Structural Model and Mediation Analysis

**Figure 2:**
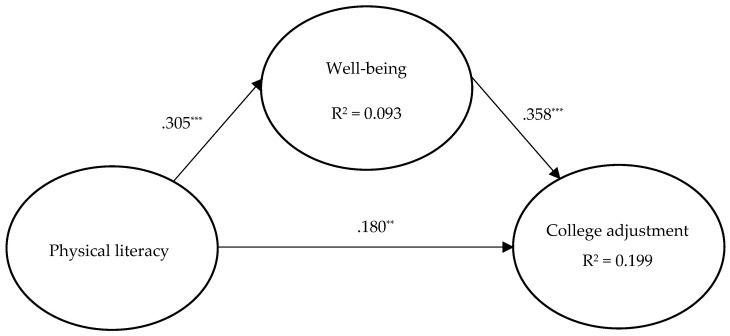
Structural model illustrating the relationships among physical literacy, well-being, and college adjustment. ** *p* < .01; *** *p* < .001.

The structural model demonstrated a good fit to the data, χ^2^(41) = 56.114, *p* = .058, χ^2^/df = 1.369, CFI = 0.996, TLI = 0.995, RMSEA = 0.036, and SRMR = 0.018. The standardized path coefficients are illustrated in Figure 2, and the detailed path analysis results are presented in Table 3. PL was positively associated with WB (B = 0.384, β = 0.305, SE = 0.076, C.R. = 5.084, *p* < .001), supporting H1. WB was positively associated with CA (B = 0.265, β = 0.358, SE = 0.043, C.R. = 6.116, *p* < .001), supporting H2. PL was also positively associated with CA (B = 0.168, β = 0.180, SE = 0.055, C.R. = 3.041, *p* = .002), supporting H3.

The indirect effect of PL on CA through WB was significant (B = 0.102, β = 0.109, bootstrap *p* = .001), supporting H4. The total effect of PL on CA was also significant (B = 0.270, β = 0.289, bootstrap *p* = .001). These findings indicate that WB partially mediated the relationship between PL and CA. PL explained 9.3% of the variance in WB, and together, PL and WB explained 19.9% of the variance in CA.

## 4. Discussion

This study examined the relationships among PL, WB, and CA among Chinese international students in South Korea who participated in basketball at least once per week. The findings showed that PL was positively associated with both WB and CA, and that WB was positively associated with CA. In addition, WB partially mediated the relationship between PL and CA. These results suggest that PL may be relevant not only to movement-related functioning but also to broader psychosocial adaptation among Chinese international students who participated in basketball at least once per week.

A central contribution of this study is that it extends current understanding of the potential relevance of PL beyond PA participation and health-related behavior. PL is commonly conceptualized as encompassing motivation, confidence, physical competence, self-expression, communication, knowledge, and understanding (Carl et al., 2022; Dudley, 2015; Edwards et al., 2017; Whitehead, 2019; Whitehead et al., 2018). These characteristics may help students engage more confidently and meaningfully in PA contexts. For Chinese international students, such engagement may be particularly important because adaptation to university life involves not only academic adjustment but also emotional coping, social relationship formation, and participation in unfamiliar cultural environments (Huang et al., 2024; Smith & Khawaja, 2011; Zhang & Goodson, 2011). In this respect, PL may be understood as a resource associated with students’ capacity to participate in, remain engaged in, and benefit from socially meaningful PA and sport contexts.

The positive association between PL and WB can be interpreted through SDT. Students with higher PL may experience stronger perceived competence in PA settings, greater confidence in their ability to participate, and more meaningful engagement with others. In group-based sport contexts such as basketball, these experiences may also support relatedness by providing opportunities for interaction, cooperation, and shared participation. These experiences are consistent with SDT, particularly the basic psychological needs for competence and relatedness, and may also support autonomy when students participate voluntarily and experience a sense of personal agency in PA settings (Deci & Ryan, 2000; Ntoumanis & Moller, 2025). Therefore, the association between PL and WB may reflect not only students’ PA participation but also the extent to which students with higher PL experience PA as meaningful, socially connected, and supportive of competence.

At the same time, the observed association between PL and WB may partly reflect personal and lifestyle characteristics associated with regular sport participation. Previous research suggests that sport participation may provide opportunities for personal and social development, including self-regulation, social connectedness, resilience, and coping with challenges or failure (Eime et al., 2013; Holt et al., 2017; Opstoel et al., 2020). These characteristics may independently contribute to higher WB and may also overlap with some dimensions of PL. Because personality traits, lifestyle organization, self-regulation, and resilience were not directly measured in the present study, it is not possible to determine whether the observed association with WB was attributable specifically to PL or partly to these related personal and lifestyle factors. Accordingly, the findings should be interpreted as evidence of an association rather than as confirmation that PL alone accounts for differences in WB.

The positive association between WB and CA further emphasizes the importance of psychological functioning in international student adaptation. International students often face academic pressure, language barriers, cultural differences, social isolation, and reduced access to familiar support networks (Andrade, 2006; Brunsting et al., 2018; Smith & Khawaja, 2011). Students with higher levels of emotional, psychological, and social WB may be better able to cope with these academic and cultural challenges, maintain positive interpersonal relationships, and engage actively with university life. Their adjustment is also shaped by contextual conditions, including social support, campus resources, inclusive environments, and opportunities for intercultural interaction and broader social engagement (Huang et al., 2024). International student adjustment may further require the ongoing reappraisal of expectations, social norms, and personal goals as students navigate unfamiliar educational and cultural contexts (J. Wang et al., 2025). Thus, WB may be viewed not only as an outcome of successful adjustment but also as a psychological resource associated with students’ capacity to navigate the demands of and engage with the host university environment (Credé & Niehorster, 2012; Diener et al., 2018; Keyes, 2002).

PL also had a significant direct association with CA after accounting for WB. This finding suggests that PL may be associated with adjustment through pathways beyond general WB. For example, students with higher PL may be more likely to participate confidently in campus-based PA, communicate with peers, develop social ties, and experience a stronger sense of belonging. One possible interpretation is that participation in basketball at least once per week provided opportunities for repeated peer interaction, cooperation, and social connection. However, because these contextual experiences were not directly measured, this explanation remains tentative. This interpretation is consistent with previous research suggesting that recreation and sport participation can support intercultural friendship, social development, and psychosocial adjustment (Glass et al., 2014; Holt et al., 2017; Opstoel et al., 2020). Thus, PL may be linked to CA both through its association with WB and through potential patterns of behavioral engagement and social integration within the campus community.

One of the most important findings of this study was the significant indirect effect of PL on CA through WB. This result suggests that WB may represent a potential psychological pathway linking movement-related competencies with broader university adaptation. From a behavioral science perspective, this finding is important because it illustrates how a multidimensional behavioral resource, PL, may be associated with successful adaptation through psychological functioning. Rather than being associated with adjustment only through the frequency of PA participation, PL may also be linked to adaptation partly through positive psychological experiences, including confidence, competence, social connection, and meaningful engagement. These experiences may be associated with higher WB, which in turn may be linked to more successful adjustment to university life (Deci & Ryan, 2000; Diener et al., 2018; Huppert & So, 2013; Keyes, 2002; Ryff, 2014).

Although the explained variance in WB (R^2^ = 0.093) and CA (R^2^ = 0.199) was modest, these findings should be interpreted within the broader context of international student adaptation research. CA is a multidimensional outcome influenced by numerous individual, interpersonal, institutional, and cultural factors, including language proficiency, acculturative stress, social support, academic demands, length of residence, and perceived belonging (Brunsting et al., 2018; Credé & Niehorster, 2012; Zhou et al., 2008). Consequently, it would be unrealistic to expect a single behavioral construct to explain a large proportion of the variance in adjustment. Rather, the present findings suggest that PL and WB may represent one meaningful psychosocial pathway among multiple factors associated with successful adaptation within this sample of Chinese international students who participated in basketball at least once per week. From an intervention perspective, this may be particularly valuable because PL is potentially modifiable through educational, recreational, and sport-based programs (Carl et al., 2022; Holt et al., 2017; Opstoel et al., 2020).

### 4.1. Theoretical and Practical Implications

Theoretically, this study extends PL research in two main ways. First, it situates PL within the context of international student adaptation rather than limiting its relevance to PA participation and health-related outcomes. Second, it identifies WB as a plausible psychological mechanism through which PL may be associated with broader university adjustment. By linking PL, WB, and CA within a single mediation model, the study extends previous PL research into the context of international student adjustment (Cairney et al., 2019; Caldwell et al., 2020; Chen, 2015; Ma et al., 2021).

The findings also indicate that SDT provides a useful interpretive framework for understanding these relationships. PL-related attributes such as confidence, perceived competence, self-expression, communication, and knowledge may help students engage more actively and meaningfully in PA contexts. Such contexts may provide opportunities to experience competence and relatedness and, when participation is self-directed, autonomy. These need-supportive experiences may be associated with higher WB and, indirectly, with more successful adjustment (Deci & Ryan, 2000; Ntoumanis & Moller, 2025). However, because psychological need satisfaction was not directly measured, this SDT-based explanation should be regarded as a theoretically grounded interpretation rather than a tested mechanism.

Practically, the findings suggest that universities may consider sport and recreation programs as potentially meaningful components of student support rather than solely as peripheral extracurricular activities. For Chinese international students, well-designed PA and sport programs may provide structured behavioral settings associated with social connection, psychological WB, and campus integration. This interpretation is consistent with recent evidence showing that international students perceive supportive people, campus resources, diverse cultural access, inclusive environments, and social events as important factors for cross-cultural adjustment (Huang et al., 2024). Programs that are beginner-friendly, socially inclusive, and culturally responsive may therefore be particularly useful for students who experience social isolation or difficulty entering existing peer networks (Glass et al., 2014; Holt et al., 2017; Huang et al., 2024).

The findings provide a rationale for universities to pilot and evaluate sport-based support strategies for international students, including introductory recreation programs for newly arrived students, peer-led basketball or team sport activities, mixed domestic–international student sport groups, and culturally responsive PA programs that reflect students’ interests and backgrounds (Glass et al., 2014; Holt et al., 2017; Huang et al., 2024). These programs could be designed not only to increase participation frequency but also to strengthen confidence, communication, perceived competence, enjoyment, and social belonging (Carl et al., 2022; Opstoel et al., 2020). Future program evaluations should determine whether such approaches actually improve PL, WB, social integration, and CA over time.

Taken together, the findings extend the emerging literature on PL by suggesting its potential relevance beyond PA participation and health-related outcomes (Cairney et al., 2019; Caldwell et al., 2020; Chen, 2015; Ma et al., 2021). Specifically, the study suggests that PL may represent a behavioral and psychosocial resource associated with successful adaptation among Chinese international students who participated in basketball at least once per week. By identifying WB as a potential partial psychological mechanism linking PL and CA, the study contributes to a more comprehensive understanding of how movement-related competencies may be associated with psychosocial adaptation in higher education settings.

### 4.2. Limitations and Future Directions

Several limitations should be acknowledged. First, the cross-sectional design precludes causal and temporal inferences. Although the mediation model was theoretically specified, the results cannot establish the direction of the relationships among PL, WB, and CA or determine whether changes in PL precede changes in WB and adjustment. Longitudinal and intervention studies are needed to examine whether changes in PL are followed by subsequent changes in WB and adjustment over time.

Second, the sample consisted only of Chinese international students in South Korea who participated in basketball at least once per week. Because the study did not include students who were physically inactive or who did not participate in basketball or other forms of regular PA, it was not possible to determine whether the observed relationships differed according to sport participation status or PA level. Future studies should include both physically active and inactive international students and examine whether sport participation or PA moderates the relationships among PL, WB, and CA.

Third, all variables were measured using self-report instruments. Although the measurement models demonstrated acceptable to good fit and reliability, self-report data may be affected by social desirability, shared method variance, and subjective interpretation of items. Future studies could incorporate objective indicators of PA, qualitative interviews, peer or program-level data, and longitudinal tracking of adjustment.

Fourth, the study did not assess several personal, lifestyle, and contextual factors that may influence WB and international student adjustment, such as personality traits, self-regulation, self-control, resilience, lifestyle organization, language proficiency, acculturative stress, perceived discrimination, social support, academic performance, and institutional support (Brunsting et al., 2018; Smith & Khawaja, 2011; Zhou et al., 2008). Some of these characteristics may be associated with regular sport participation and may partly explain the observed relationships among PL, WB, and CA. Future research should incorporate these variables, together with academic year, length of study in South Korea, and frequency of sport participation, to distinguish the specific contribution of PL from related personal, behavioral, and contextual influences and to develop a more comprehensive model of international student adaptation.

Finally, the study focused on basketball participation as one PA context. Although basketball may provide meaningful opportunities for teamwork, communication, and social connection, these features were not directly assessed in the present study. Future studies should compare different types of PA contexts, such as individual exercise, recreational sport clubs, fitness programs, and culturally specific physical activities. Such research could clarify whether the psychosocial associations observed in this study are specific to team sport settings or generalizable across different movement contexts.

## 5. Conclusions

This study examined the relationships among PL, WB, and CA among Chinese international students in South Korea who participated in basketball at least once per week. The results showed that PL was positively associated with WB, supporting H1, and that WB was positively associated with CA, supporting H2. PL was also positively associated with CA, supporting H3, while WB partially mediated the relationship between PL and CA, supporting H4. Thus, all four hypotheses were supported.

These findings suggest that PL may represent a multidimensional behavioral and psychosocial resource associated with university adjustment among Chinese international students who participate in basketball at least once per week. In particular, PL was associated with CA both directly and indirectly through its association with WB. This highlights psychological functioning as a potential pathway connecting movement-related competencies with broader psychosocial adaptation.

The study contributes to behavioral science by extending PL research into the context of Chinese international student adjustment. It also suggests that campus-based PA and sport programs may have potential relevance for the adjustment and WB of Chinese international students, particularly those who participate in organized sport. Future longitudinal and intervention-based studies are needed to clarify causal pathways and determine whether the present findings can be replicated among more diverse groups of international students.

## Figures and Tables

**Table 1:** Descriptive statistics and correlations among the study variables (*N* = 288).

Variable	Mean	SD	Skewness	Kurtosis	1	2	3
1. Physical literacy	3.70	0.95	−1.11	−0.50	—		
2. Well-being	4.69	1.18	−1.70	1.14	.298 ***	—	
3. College adjustment	3.71	0.90	−1.22	−0.39	.275 ***	.400 ***	—

*** *p* < .001.

**Table 2:** Discriminant validity based on the Fornell–Larcker criterion.

Construct	1	2	3
1. Physical literacy	**.760**	.298	.275
2. Well-being	.298	**.840**	.400
3. College adjustment	.275	.400	**.730**

Note. Diagonal values in bold represent the square root of the AVE; off-diagonal values represent correlations among constructs.

**Table 3:** Structural path analysis results.

Path	B	β	SE	C.R.	*p*
PL → WB (H1)	0.384	0.305	0.076	5.084	<.001
WB → CA (H2)	0.265	0.358	0.043	6.116	<.001
PL → CA (H3)	0.168	0.180	0.055	3.041	.002
Indirect effect (PL → WB → CA) (H4)	0.102	0.109	0.034 ^†^	—	.001 ^†^
Total effect (PL → CA)	0.270	0.289	0.067 ^†^	—	.001 ^†^

Note. PL = physical literacy; WB = well-being; CA = college adjustment; C.R. = critical ratio. ^†^ Bootstrap standard error and two-tailed *p* values are based on 2000 bootstrap resamples.

## Data Availability

The data presented in this study are available on request from the corresponding authors due to privacy and ethical restrictions.

## Abbreviations

The following abbreviations are used in this manuscript:

WB	well-being
CA	college adjustment
PA	physical activity
PL	physical literacy
SDT	Self-Determination Theory
SEM	structural equation modeling
CFA	confirmatory factor analysis
CFI	comparative fit index
TLI	Tucker–Lewis index
RMSEA	root mean square error of approximation
SRMR	standardized root mean square residual
CR	composite reliability
AVE	average variance extracted
